# An approach for predicting the compressive strength of cement-based materials exposed to sulfate attack

**DOI:** 10.1371/journal.pone.0191370

**Published:** 2018-01-18

**Authors:** Huaicheng Chen, Chunxiang Qian, Chengyao Liang, Wence Kang

**Affiliations:** 1 School of Materials Science and Engineering, Southeast University, Nanjing, Jiangsu province, China; 2 Research Institute of Green Construction Materials, Southeast University, Nanjing, Jiangsu province, China; East China Normal University, CHINA

## Abstract

In this paper, a support vector machine (SVM) model which can be used to predict the compressive strength of mortars exposed to sulfate attack was established. An accelerated corrosion test was applied to collect compressive strength data. For predicting the compressive strength of mortars, a total of 638 data samples obtained from experiment was chosen as a dataset to establish a SVM model. The values of the coefficient of determination, the mean absolute error, the mean absolute percentage error and the root mean square error were used for evaluating the predictive accuracy. The main factors affecting the predicted compressive strength were obtained by sensitivity analysis. A SVM model was calibrated, validated, and finally established. Moreover, the performance of the SVM model was compared to an artificial neural network (ANN) model. Results show that the prediction values from the SVM model were close to the experimental values; the main factors sensitive to concrete compressive strength were exposure time, water-cement ratio and sulfate ions; the performance of the SVM model was better than the ANN model. The SVM model developed in this study can be potentially used for predicting the compressive strength of cement-based materials servicing in harsh environments.

## Introduction

Concrete is an important structural material being used in civil engineering and industrial facilities. The strength of concrete is considered as one of the most important property for a given concrete mix design. Besides the constituent of materials, the strength is also affected by environmental exposure and extreme working conditions [1]. In harsh environments, especially the areas with abundant sulfate ions, the properties of concrete materials can easily deteriorate, which could affect the safety of engineering structures [2]. For the safety assessment of existing structures, compressive strength is often considered as the most important indicator of concrete quality [3, 4]. Monitoring concrete strength during service can give an idea about the time for concrete quality control and performance maintenance [5]. In addition, predicting concrete strength can be helpful in assessing the deterioration of concrete structures and increasing their safety [6]. Thus, methods for predicting and estimating real-time concrete strength are important. Unfortunately, due to the complex degradation mechanisms and multiple influencing parameters [7], there is no effective method to predict compressive strength of cement-based materials in harsh environments.

To date, there are two categories about the prediction of concrete compressive strength [8]. The first category is traditional mathematics statistical forecasting methods. It needs a huge amount of data. When the sample data tends to infinity, it tends to predict real results, but the actual number of samples is often limited. The second category, nonlinear prediction methods, lacks a unified mathematical theory. The predicted results are often a partial optimal solution, rather than a global optimal solution. For conventional concrete, the above categories can all be used to predict the values of compressive strength, but for concrete exposed to sulfate attack as the number of input factor increases, the relationship between the input factors and the compressive strength becomes highly nonlinear and complex. Hence, the regression models are not suitable for predicting the values of compressive strength of concrete in harsh environments. Therefore, more attentions have been paid to models based on artificial intelligence. Machine learning techniques, such as artificial neural network (ANN) is increasingly used to simulate the strength of concrete materials and has become an important research area [9–12].

An ANN model is usually consisted of inputs, weights, sum function, activation function and outputs. The related algorithms require setting up of different learning parameters, the optimal number of nodes in the hidden layer and the number of hidden layers. Until now, back-propagation (BP) algorithm, which adjusts connection weights and bias values during training, has been widely used for training an ANN model. However, ANN models still have disadvantages: (1) The information about the relative significance of the various parameters cannot be provided [13]; (2) A reasonable interpretation of the overall structure of the network is hard to be established [14]. In addition, ANN models have some intrinsic disadvantages such as slow convergence speed, less generalizing effectiveness, arriving at local minimum and over-fitting problems [15]. To overcome those limitations, in recent years, researchers have explored the potential of support vector machine (SVM) in performance of cement-based materials.

SVM, a nonlinear modeling approach, proposed based on the statistical theory by Vapnik [16] is being applied in the field of civil engineering. Unlike ANN models, a SVM model has the advantage of reducing training error and being a unique and globally optimum [17]. The method has excellent generalization capability when solving non-linear problems. It can also overcome the problem of small sample size. For example, Yan and Shi [18] used SVM to predict the elastic modulus of normal and high strength concrete. The analytical results showed that the SVM outperformed other models. Chou et al. [19] predicted the compressive strength of high performance concrete by using the SVM technique, and the behavior simulation capability of SVM was investigated using concrete data from several countries. Cheng et al. [20] proposed an advanced hybrid AI model that fused fuzzy logic, weight SVM and fast messy genetic algorithms to predict compressive strength of concrete. Gupta [21] investigated the potential use of SVM for predicting CCS by combining radial basis function with SVM.

There have been few studies on the prediction of the compressive strength of cement-based materials exposed to sulfate attack using SVM. Most of these studies established the prediction models of concrete compressive strength mainly based on the material factors (e.g., water-binder ratio, water content and aggregate content) and curing age [9–12, 18–20]. However, for concrete subjected to service in a harsh environment, environmental factors are of great importance to the compressive strength and should be considered in the compressive strength prediction models.

To accurately predict the concrete compressive strength using SVM, a data set with a large amount of experimental data is required. Compared to field testing, the indoor accelerated corrosion testing can be performed with a controlled environment and thus has been widely applied to quickly obtain the compressive strength of concrete [22, 23].

In this study, support vector machine was applied to predict the compressive strength of cement-based materials exposed to sulfate attack. To establish a SVM model for predicting compressive strength, the accelerated corrosion test of mortars with different water-cement (w/c) ratios was carried out, and 638 sets of data from our experiment was collected. The SVM model was first calibrated and then validated. The values of the coefficients of determination (R^2^), the mean absolute error (MAE), and the mean absolute percentage error (MAPE) and the root mean square error (RMSE) were used for evaluating the predictive accuracy. Furthermore, the main factors that influence the predicted compressive strength were obtained by sensitivity analysis. Finally, the performance of the SVM model was further evaluated by comparison with an ANN model.

## Methodology

### 2.1 Theory of SVM

The essence of SVM is to map data samples with highly nonlinear relationships in the low-dimensional space onto a high-dimensional space. The data samples are classified according to the principle of risk structure optimization. The regression function *f* (*x*, *w*) is expressed by the following equation:

$$f(x,\omega )=\sum_{j=1}^{n}\omega_{j}*g_{j}(x)+b$$ (1)

where *g*_*j*_(*x*) is a mapping function, *ω*_*j*_ is the weight coefficient, *b* is the threshold.

According to the principle of structural risk minimization, regression optimization constraints can be expressed as:

$$\text{min}\frac{1}{2}{\left\|\omega \right\|}^{2}+c\sum_{i=1}^{n}\left(\varepsilon_{i}+\varepsilon_{j}\right)$$ (2)

$$\text{subject}\;\text{to}\;\{\begin{array}{c} y_{i}-f\left(x_{i},\omega \right)\leq \varepsilon +\varepsilon_{i}^{*}\\ f\left(x_{i},\omega \right)-y_{i}\leq \varepsilon +\varepsilon_{i}^{*}\\ \varepsilon_{i},\varepsilon_{i}^{*}\gg 0,i=1\ldots \ldots \ldots n \end{array}$$

where *c* is the penalty parameter, *ε*_*i*_ and $\varepsilon_{i}^{*}$ are the slack variables, *ε* is the insensitive loss function.

Then, the optimization problem can be transformed into a dual problem and the regression function can be written as:

$$f(x,\;w)=\sum_{i=1}^{n_{sv}}\left(\alpha_{i}-\alpha_{i}^{*})k(x,x_{i}\right)+b$$ (3)

$$\text{subject}\;\text{to}:\;0\leq \alpha_{i}^{*}\leq c,0\leq \alpha_{i}\leq c$$

where *n*_*sv*_ is the number of support vectors; *k*(*x*, *x*_*i*_) is the kernel function; *α*_*i*_ and ${\;\alpha }_{i}^{*}$ are the Lagrange multipliers; *c* is the penalty parameter. $\alpha_{i}^{*}$ can be obtained by solving the above constrained optimization problem. Threshold value *b* can be calculated by $\alpha_{i}^{*}$.

### 2.2 Calculation process

**Fig 1:**
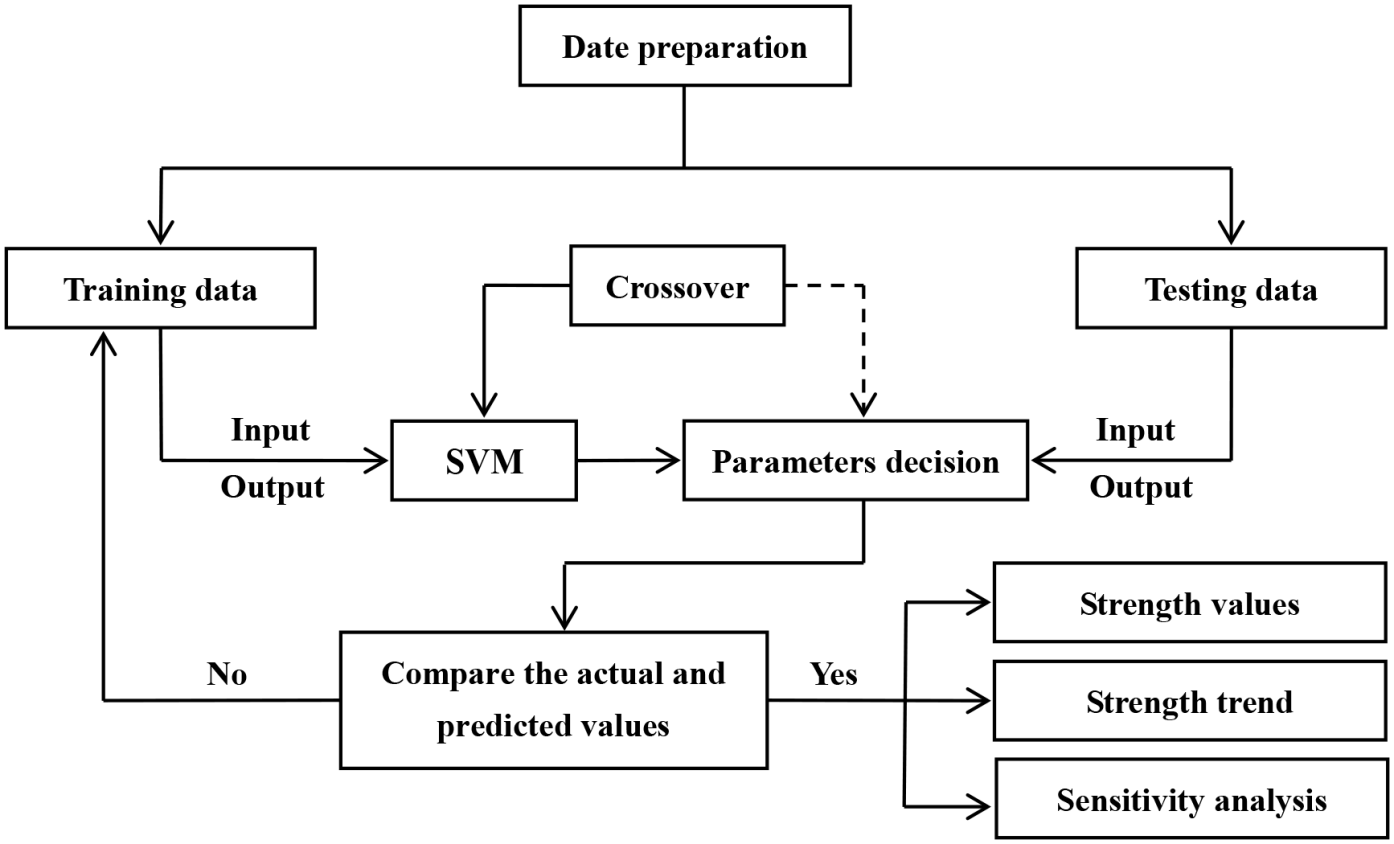
Calculation process of SVM.

The MATLAB software was used to implement the SVM model. The calculation process of the SVM model is shown in Fig 1.

1. Determine the training dataset
  $$\text{T}=\left\{\left(x_{1},y_{1}\right),\cdots ,\left(x_{l},y_{l}\right)\right\}\in (\text{X}\times \text{Y}{)}^{l}$$ (4)
  where, {*x*_*i*_, *y*_*i*_}_*i* = 1_, *x*_*i*_ ∈ *R*^*n*^ is the factor of influencing the compressive strength of group *i*; *y*_*i*_ ∈ *R*^*n*^ is the expected output intensity value of group *i* from the training data.
2. Choose the appropriate kernel function and solve the optimization problem
  In this study, radial basis function (RBF) is chosen for the kernel function:
  $$\text{K}\left(\text{x},x_{i}\right)=\text{exp}\left(-\text{gamma}{\left\|x-x_{i}\right\|}^{2}\right)$$ (5)
  According to the principle of structure optimization, the regression optimization goal is expressed as:
  $$min^{\alpha }\frac{1}{2}\sum_{i=1}^{j}\sum_{j=1}^{l}y_{i}y_{j}\alpha_{i}\alpha_{j}\text{exp}\left(-\text{gamma}{\left\|x_{i}-x_{j}\right\|}^{2}\right)-\sum_{j=1}^{l}\alpha_{j}$$ (6)
  $$\text{subject}\;\text{to}\sum_{i=1}^{l}y_{i}\alpha_{i}=0,\;0\leq \alpha_{i}\leq c,\;i=1,2,\ldots ,l$$
  Then, the optimal solution can be calculated as:
  $$\alpha^{*}={\left(\alpha_{1}^{*},\ldots ,\alpha_{l}^{*}\right)}^{T}$$ (7)
  where *c* is the penalty parameter; -gamma (*g*) is the kernel function parameter.
3. Optimize the parameter
  The punishing of parameter *c* and kernel function parameter *g* would directly affect the prediction results. However, there is so far no a best way to determine the *c* and *g*. In this study, the parameters *c* and *g* were obtained by a *K*-fold crossover algorithm [24]. All the average prediction accuracy is CV. When CV achieves the best accuracy, *c* and *g* are the optimum parameters. Then the optimal solution a* was solved according to the type of the optimal constraint.
4. Calculate the threshold value
  The threshold b* is calculated by the equation as follow:
  $$b^{*}=y_{j}-\sum_{i=1}^{l}y_{i}\alpha_{i}^{*}K(x_{i}-x_{j})\text{exp}\left(-\text{gamma}{\left\|x_{i}-x_{j}\right\|}^{2}\right)$$ (8)
5. Construct decision function
  After parameters a*, b* and *g* having been determined, further calculation is carried out as follow:
  $$f(x)=sgn(\sum_{i=1}^{l}\omega_{i}\text{exp}\left(g{\left\|x_{i}-x\right\|}^{2}\right)+b^{*})$$ (9)
  where *x* is the prediction data.

### 2.3 Performance evaluation methods

The R^2^, MAE, MAPE and RMSE were used to evaluate the prediction accuracy of the SVM model [25]. R^2^ is a measure of how well the independent variables approximate the measured dependent variable, while MAE, MAPE and RMSE are used as a measure of differences between the values predicted by the model. Low values of MAE, MAPE and RMSE, and high values of R^2^ are generally indicative of a good performance. They are defined as follows:

$$R^{2}=\frac{{\left(n\sum y_{i}y-\sum y_{i}\sum y\right)}^{2}}{\left(n\sum {y_{i}}^{2}-(\sum {y_{i}}^{2}))\;(n\sum y^{2}-{\left(\sum y\right)}^{2}\right)}$$ (10)

$$\text{MAE}=\frac{1}{n}\sum_{i=1}^{n}\left|y-y_{i}\right|$$ (11)

$$\text{MAPE}=\frac{1}{n}\sum_{i=1}^{n}\left|\frac{y-y_{i}}{y}\right|$$ (12)

$$\text{RMSE}=\sqrt{\frac{1}{n}\sum_{i=1}^{n}{\left(y-y_{i}\right)}^{2}}$$ (13)

where *y* and *y*_*i*_ are the actual value and predicted value, respectively; *n* is the number of data samples.

## Experimental program

### 3.1 Materials

Type II ordinary Portland (P II 52.5) cement purchased from China United Cement Corporation was used in this study. The chemical composition and mineral original composition of cement are shown in Tables 1 and 2, respectively. ISO standard sand with a density of 2.58 g/cm^3^ obtained from Xiamen standard sand Co., Ltd. was used as fine aggregate. The maximum sizes of the sands were 4.75 mm. A superplasticizer (SP) with 36.8 wt% solid content was purchased from Jiangsu Sobute New materials Co. Ltd. and its water-reducing ratio was 29.8%. Tap water was used in concrete mixtures and curing application in this study. Na_2_SO_4_, analytically pure, obtained from China Guoyao Chemical Company, is used to prepare different concentrations of sodium sulfate solutions.

**Table 1:** The chemical composition of cement (%).

Type	CaO	SiO_2_	Al_2_O_3_	Fe_2_O_3_	MgO	SO_3_	K_2_O	Na_2_O	other	Loss on ignition
Cement	64.47	20.34	4.83	3.41	2.09	2.01	0.75	1.34	0.76	1.03

**Table 2:** The mineral original composition of cement (%).

C_3_S	C_2_S	C_3_A	C_4_AF	Gypsm	other
55.8	19.2	6.7	10.4	5.3	2.6

### 3.2 Mix proportion and specimens preparation

Three types of mortar (*M65*, *M50*, *M28*) with respective w/c ratio of 0.65, 0.50 and 0.28 were designed and the compositions is shown in Table 3. After the fresh concrete was prepared, the mixtures were cast into 40 × 40 × 160 mm steel moulds and compacted on a vibrating table. The samples were demoulded 24 hours after casting. After demoulding, the specimens were cured in a curing room (Temperature = 20 ± 2°C, RH > 95%) for 90 days.

**Table 3:** Mixing proportions of mortar and corrosion solutions.

Type	w/c	Cement/g	Water/g	Sand/g	SP/g	Sulfate/wt%
*M65-S1*	0.65	385	250	1500	0	2%
*M65-S2*	0.65	385	250	1500	0	10%
*M65-S3*	0.65	385	250	1500	0	16.3%
*M50-S1*	0.50	450	225	1450	0	2%
*M50-S2*	0.50	450	225	1450	0	10%
*M50-S3*	0.50	450	225	1450	0	16.3%
*M28-S1*	0.28	680	190	1350	1.8	2%
*M28-S2*	0.28	680	190	1350	1.8	10%
*M28-S3*	0.28	680	190	1350	1.8	16.3%

### 3.3 Accelerated deterioration test

After curing for 90 days, the specimens were degraded in a constant temperature and constant humidity box (Shanghai Jinghong Experimental Equipment Co., Ltd.), which temperature range is from 20°C to 80°C, and humidity range is from 50% to 95%. Three different concentrations of sodium sulfate (2%, 10%, 16.3%) were used for the corrosion solutions. To accelerate the deterioration process, a dry-wet circulation method was also adopted. According to Chinese national standard GB/T 749–2008 [26], the dry-wet circulation test is that all the cured concrete specimens were fully immersed in the corrosion solutions for 8 hours at 20±2°C and then dried for 16 hours at 50±2°C in a relative humidity of 60%.

### 3.4 Measurement of compressive strength

A uniaxial compression test was carried out to measure the compressive strength of specimens. The test was carried out according to the Chinese national standard GB/T 50081–2002 [27]. It was performed by using a Material Testing Simulation machine (Wuxi Jianyi Instrument and Equipment Co., Ltd.) press with a capacity of 300 kN in compression and was carried out at a rate of 2.4 kN/s using three specimens for each deterioration age.

## Results and discussion

### 4.1 Compressive strength after deterioration

**Fig 2:**
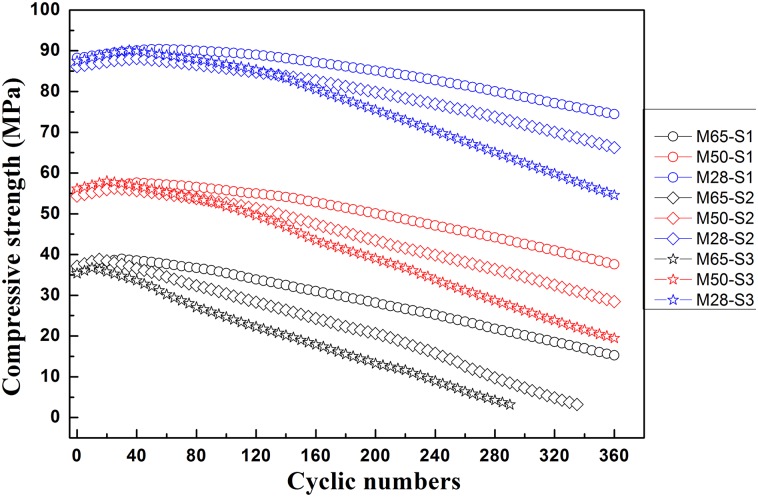
Relationship between the compressive strength and cyclic numbers.

The compressive strengths of mortars exposed to sulfate attack are shown in Fig 2. The results show that the compressive strength values of all specimens gently increased in the early stages. This increase is probably because sulfate ions diffuse slowly into specimens, and the specimens have not been corroded by the sulfate ions. When sulfate ions start to react with cement hydration products to form expanded products (gypsum and ettringite) with larger volumes than the reactants, the pore structures become denser due to the increasing volume of the expanded products in the early stage of deterioration [28]. With the increasing amounts of expanded products, the compressive strength values decreased gradually. Fig 2 also shows that, the greater the w/c ratios, the larger the descent rates of compressive strength. These different decreasing rates of compressive strength can be caused by the different sulfate resistances of specimens with different micro-pores characteristics and porosity values. The sulfate resistance is higher with a lower w/c ratio. For mortar with a lower w/c ratio, the pore structure is much denser, and the ion diffusivity is lower, and thus the amounts of sulfate ions reacting with the hydration products are smaller.

### 4.2 Data preprocessing

To successfully develop a SVM model to predict the compressive strength, sufficient experimental data is needed. In this study, the experimental data set was collected from laboratory test and 638 sample data were prepared. A total of 550 sample data were used for model training, and 88 sample data were used for model testing. The database examples are shown in Table 4.

**Table 4:** Database examples.

w/c	C/%	W/%	S/%	SO_4_^2-^/wt%	Wet-T/°C	Wet-t/h	Dry-T/°C	Dry-t/h	Exp-t/d	Fm/MPa
0.65	18	12	70	2	20	8	50	16	0	36.51
0.65	18	12	70	2	20	8	50	16	180	29.59
0.65	18	12	70	10	20	8	50	16	60	34.53
0.65	18	12	70	10	20	8	50	16	240	15.76
0.65	18	12	70	16.3	20	8	50	16	90	25.97
0.65	18	12	70	16.3	20	8	50	16	180	15.54
0.50	21.18	10.59	68.23	2	20	8	50	16	60	57.18
0.50	21.18	10.59	68.23	2	20	8	50	16	240	47.13
0.50	21.18	10.59	68.23	10	20	8	50	16	90	53.18
0.50	21.18	10.59	68.23	10	20	8	50	16	300	34.52
0.50	21.18	10.59	68.23	16.3	20	8	50	16	10	57.02
0.50	21.18	10.59	68.23	16.3	20	8	50	16	150	45.11
0.28	30.63	8.55	60.81	2	20	8	50	16	50	90.36
0.28	30.63	8.55	60.81	2	20	8	50	16	210	84.58
0.28	30.63	8.55	60.81	10	20	8	50	16	110	85.21
0.28	30.63	8.55	60.81	10	20	8	50	16	290	72.87
0.28	30.63	8.55	60.81	16.3	20	8	50	16	160	80.52
0.28	30.63	8.55	60.81	16.3	20	8	50	16	330	58.47
**…**	**…**	**…**	**…**	**…**	**…**	**…**	**…**	**…**	**…**	**…**

Notes: w/c: water-cement ratio, C: cement content, %; W: water content, %; S: sand content, %; SO_4_^2-^, sulfate concentration, wt%; Wet-T, wetting temperature, °C; Wet-t: wetting time, h; Dry-T, drying temperature, °C; Dry-t: drying time, h; Exp-t: exposure time, days; Fm: ultimate compressive strength, MPa.

The training data set was used to calibrate the model with 10 input variables, and the testing data set was used to estimate the model’s performance. As shown in Table 4, the following input variables were used: w/c ratio, cement content (C, %), water content (W, %), sand content (S, %), sulfate ions concentration (SO_4_^2-^, wt%), wetting temperature (Wet-T, °C), wetting time (Wet-t, hours), drying temperature (Dry-T, °C), drying time (Dry-t, hours), and exposure time (Exp-t, days). Ultimate compressive strength (Fm, MPa) was used as the output parameter.

### 4.3 Optimum parameters determining

**Fig 3:**
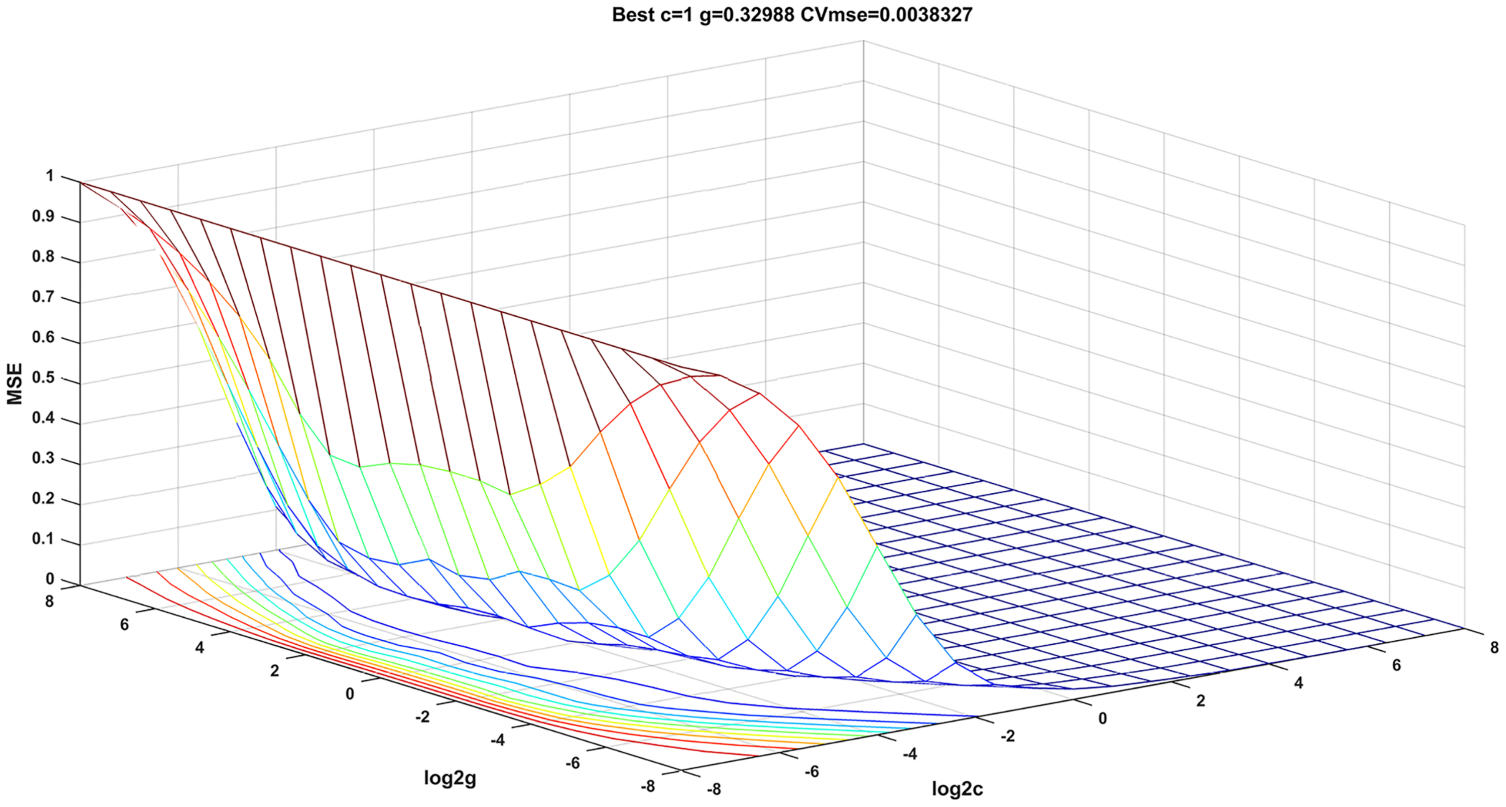
Results of parameter optimization.

As shown in Fig 3, the mean square error (MSE) was calculated by crossover operation training in the search range of *c* and *g* (search range: 2^−8^ ~ 2^8^). When the CVmse achieved 0.0038, the optimal penalty parameter *c* was 1, and the optimum parameter of the radial basis kernel *g* was 0.3299. According to the report of Yassi and Moattar [24], a smaller *c* and *g* will cause under-fitting, while a bigger *c* and *g* will cause over-fitting. Both of them will affect the generalization ability of the model.

### 4.4 Prediction performance

**Fig 4:**
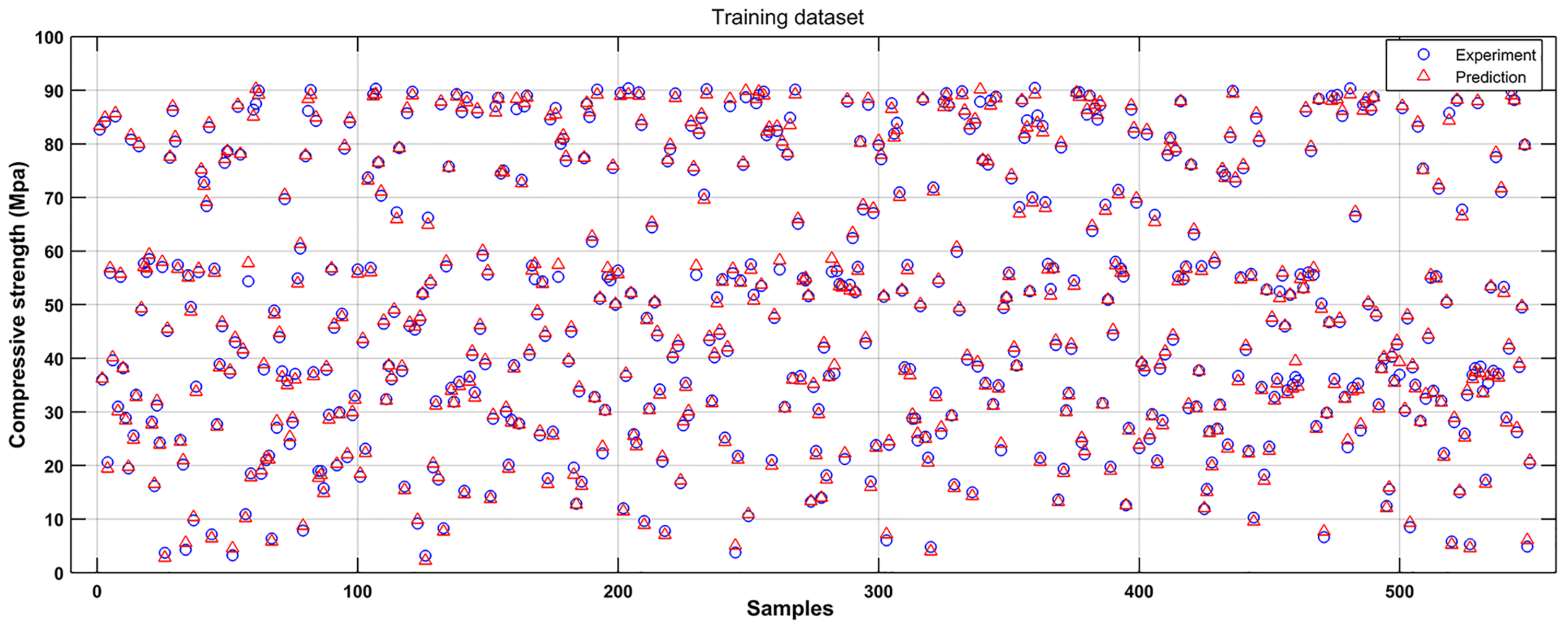
Comparison of experimental results to predicted results of SVM (Training dataset).

**Fig 5:**
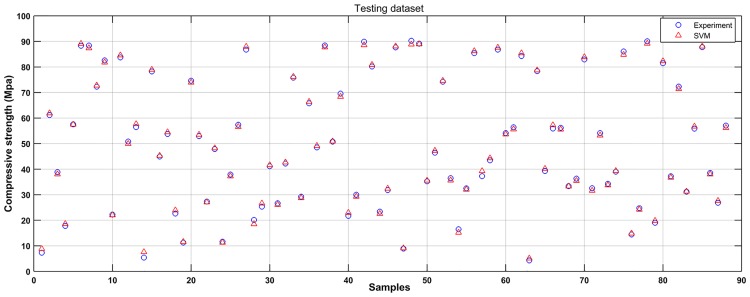
Comparison of experimental results to predicted results of SVM (Testing dataset).

**Fig 6:**
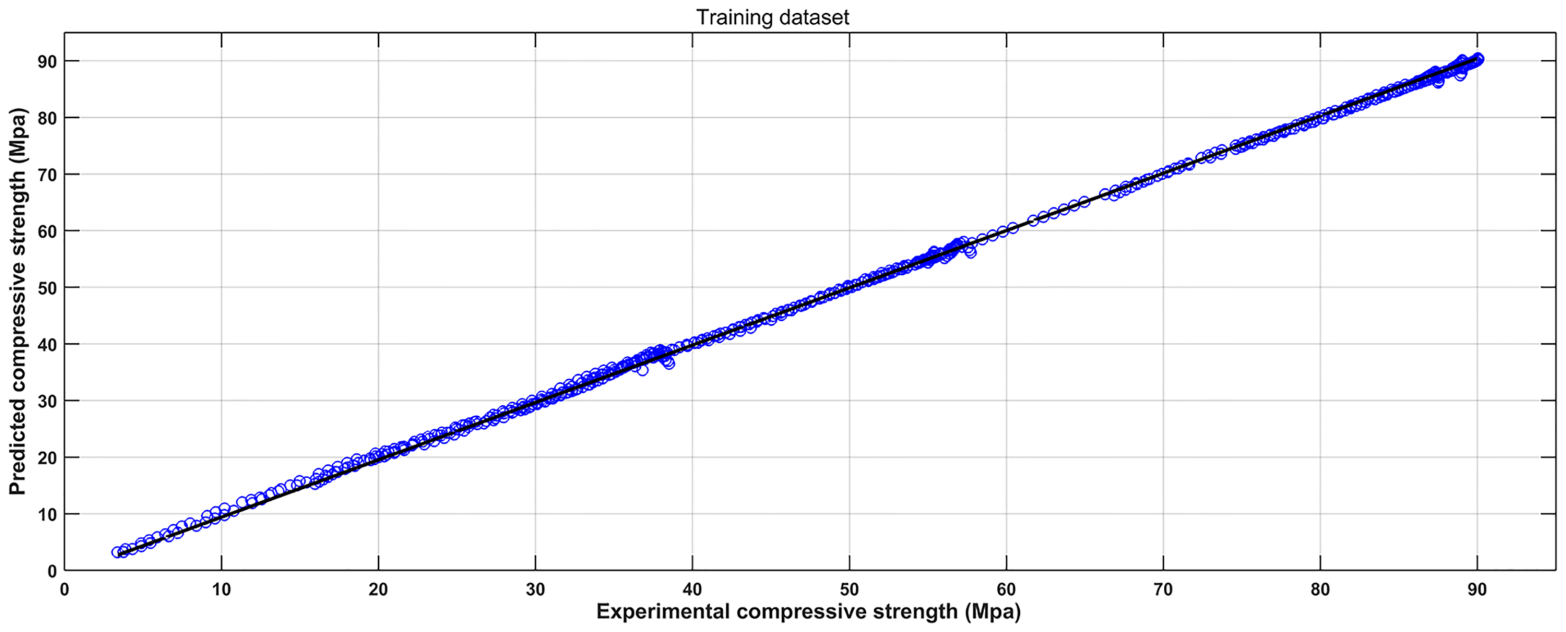
Observed versus predicted compressive strength produced by the SVM model (Training dataset).

**Fig 7:**
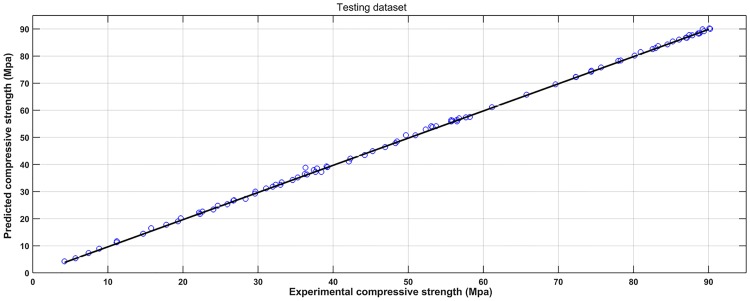
Observed versus predicted compressive strength produced by the SVM model (Testing dataset).

Based on the experimental data, a SVM model was established to learn the complicated interrelationships between compressive strength and varied input variables. For convenient comparison purposes, the scatter diagrams of the experimental and predicted results are plotted in Figs 4 and 5. It can be seen that most predicted points are close to the experimental values. Figs 6 and 7 show the observed versus predicted compressive strength produced by the SVM model. Results show that both the computational values of training data and testing data fitted well with their corresponding experimental values. Moreover, the R^2^, MAE, MAPE and RMSE between the experimental and computational results can be calculated by Eqs (10), (11), (12) and (13). The coefficient of determination of training data was 0.9994, while the coefficient of determination of testing data was 0.9991. The MAE were all less than 3.1 MPa, the MAPE were all less than 3.8%, and the RMSE of training data and testing data were less than 3.6 MPa. These results indicate that the SVM model has a good performance in predicting compressive strength of mortars exposed to sulfate attack.

### 4.5 Sensitivity analysis

**Fig 8:**
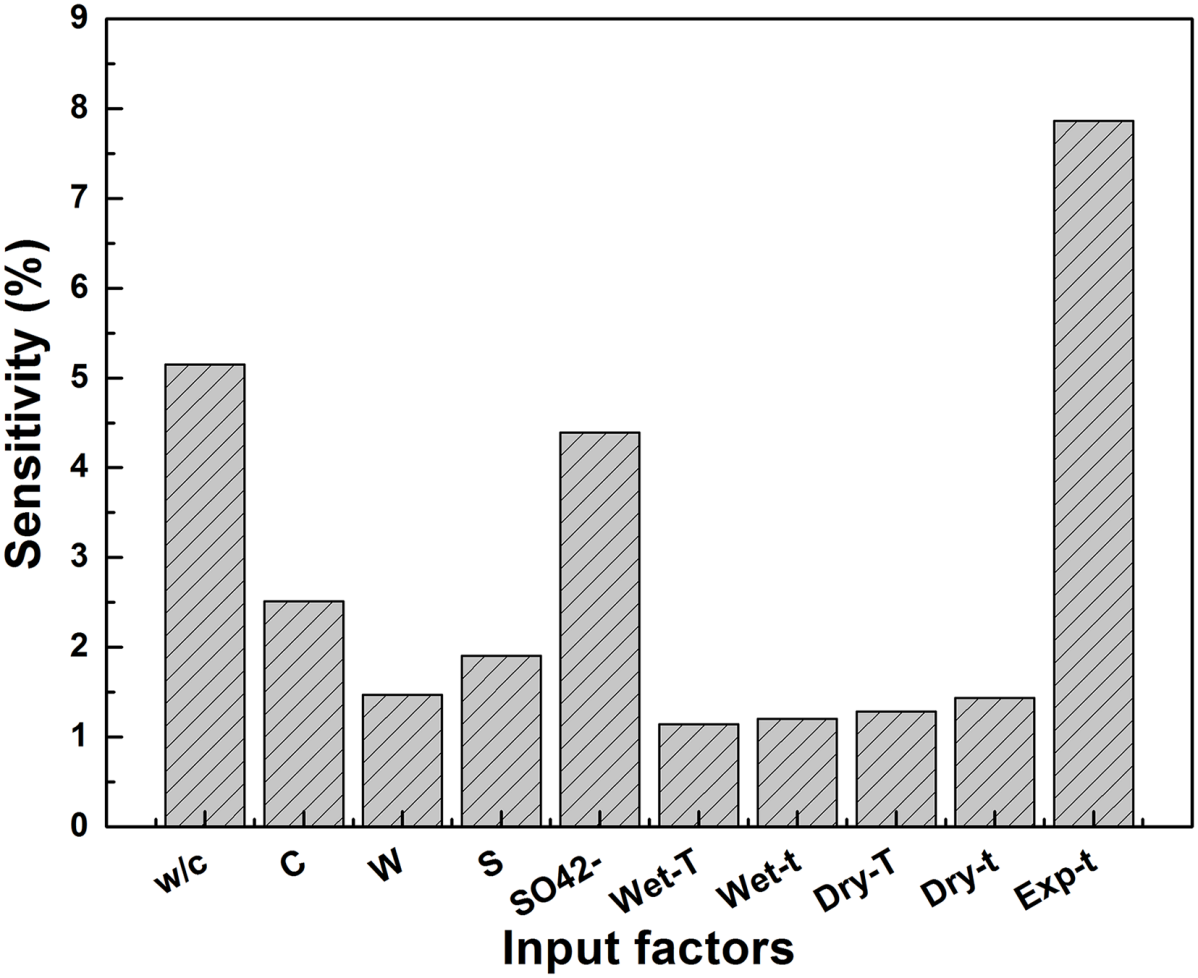
The sensitivity of the input factors. w/c: water-cement ratio, C: cement content, %; W: water content, %; S: sand content, %; SO_4_^2-^, sulfate concentration, wt%; Wet-T, wetting temperature, °C; Wet-t: wetting time, h; Dry-T, drying temperature, °C; Dry-t: drying time, h; Exp-t: exposure time, days.

To assess the changes in the output caused by the input changes of the SVM model, a sensitivity analysis was performed. According to the calculation method developed by Liong et al., the sensitivity of an input parameter can be calculated by the following formula [29].

$$S(\%)=\frac{1}{n}\sum_{j=1}^{n}{\left(\frac{\%change\;in\;output}{\%change\;in\;input}\right)}_{j}$$ (14)

where *n* is the number of data points. The sensitivity analysis was carried out on the model by varying each of input parameters, one at a time, at a constant rate of 20%, while the other input parameters were maintained. The greater the variation that is observed in the output means that greater sensitivity is presented with respect to the input value. The sensitivity analysis results are shown in Fig 8. Results show that the exposure time, w/c ratio and sulfate concentration were found to be sensitive to the predicted compressive strength. Within these factors, the exposure time was the most sensitive factor that affects the predicted compressive strength.

### 4.6 Comparison with ANN model

#### 4.6.1 Training of ANN

A comparative study between the SVM model and ANN model was carried out. An ANN is composed of many artificial neurons which are linked together via network of weights and biases, carrying the output of one neuron as input to another neuron. The training procedure of ANN is consisted of finding the optimum values of these weights and biases. One of the most useful algorithms for training a multilayer perceptron neural network is BP algorithm [30–33]. This method calculates the error between the network outputs and desired targets and propagates back to the network through a learning mechanism. As a result, the weights and biases (thresholds) are updated until the network reaches a predefined performance goal.

**Fig 9:**
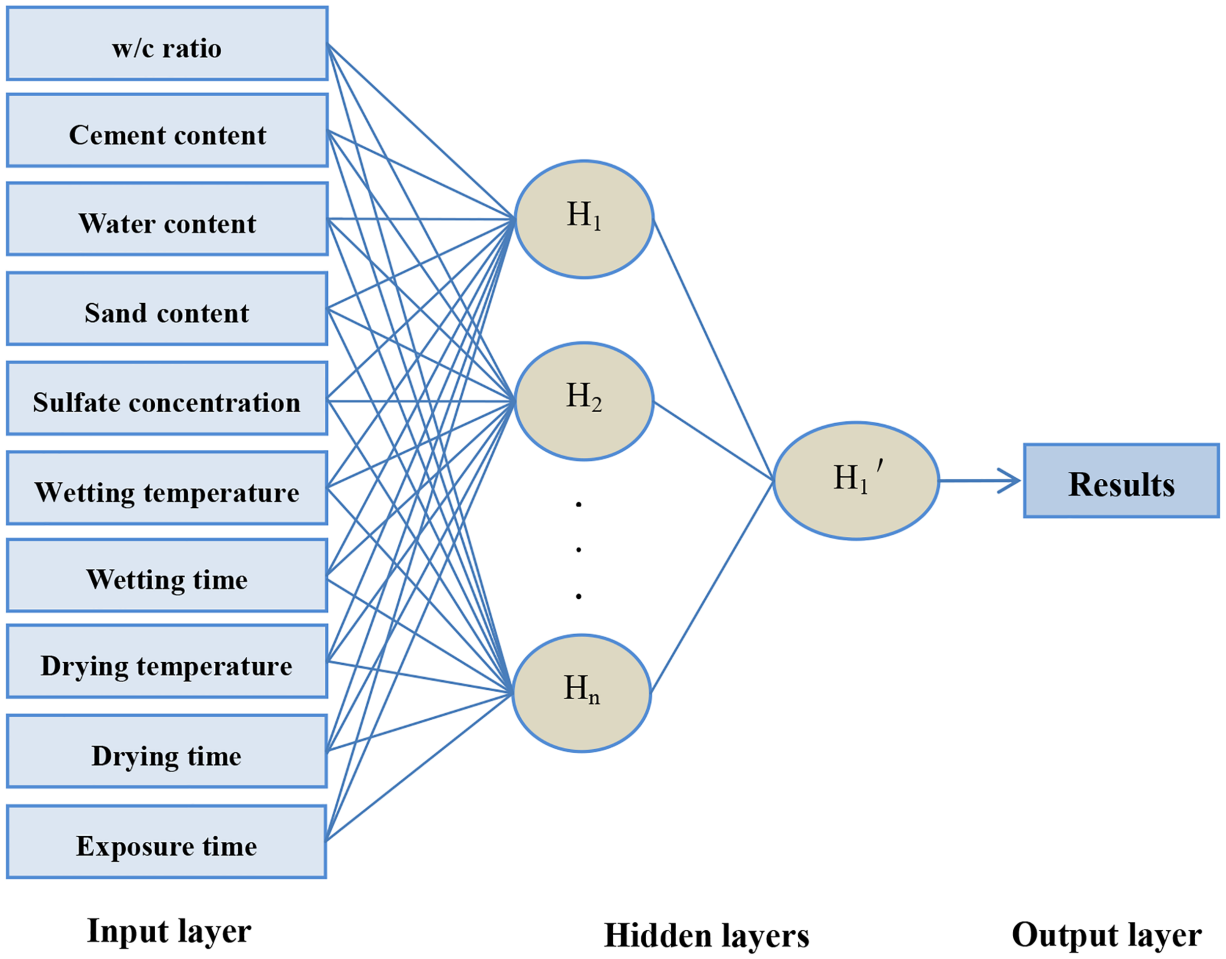
A multi-layer ANN schematic.

In this study, a BP algorithm was used to establish the ANN model. The whole operation is demonstrated in Fig 9. It can be divided into six steps. Step 1: Input training factors. Ten influencing factors are inputted into the model; Step 2: Hidden nodes calculate the output. This is a quite complex process that detailed calculated algorithm is invisible; Step 3: Output nodes calculate outputs. Step 4: Comparison of the outputs with targets and figure out the difference; Step 5: Adjust the model parameters on the basis of training rule using the results of Step 4. Calculate every values in hidden nodes in this step; Step 6: The results of step 5 are used to carry on a second training until the error is suitably small. The specific parameters are as follows:

net.trainParam.show=10;

net.trainParam.lr=0.01;

net.trainParam.mc=0.9;

net.trainParam.epochs=10000;

net.trainParam.goal=0.0001.

**Fig 10:**
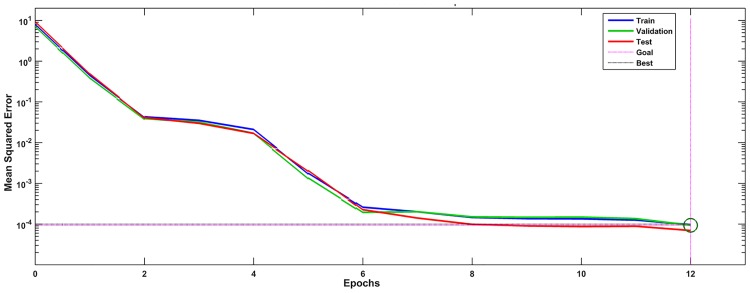
The neural network training process.

As shown in Fig 10, the training errors decreased to planned target (0.0001) at 12th epoch and it converged at that epoch.

#### 4.6.2 Results comparison between SVM and ANN

**Fig 11:**
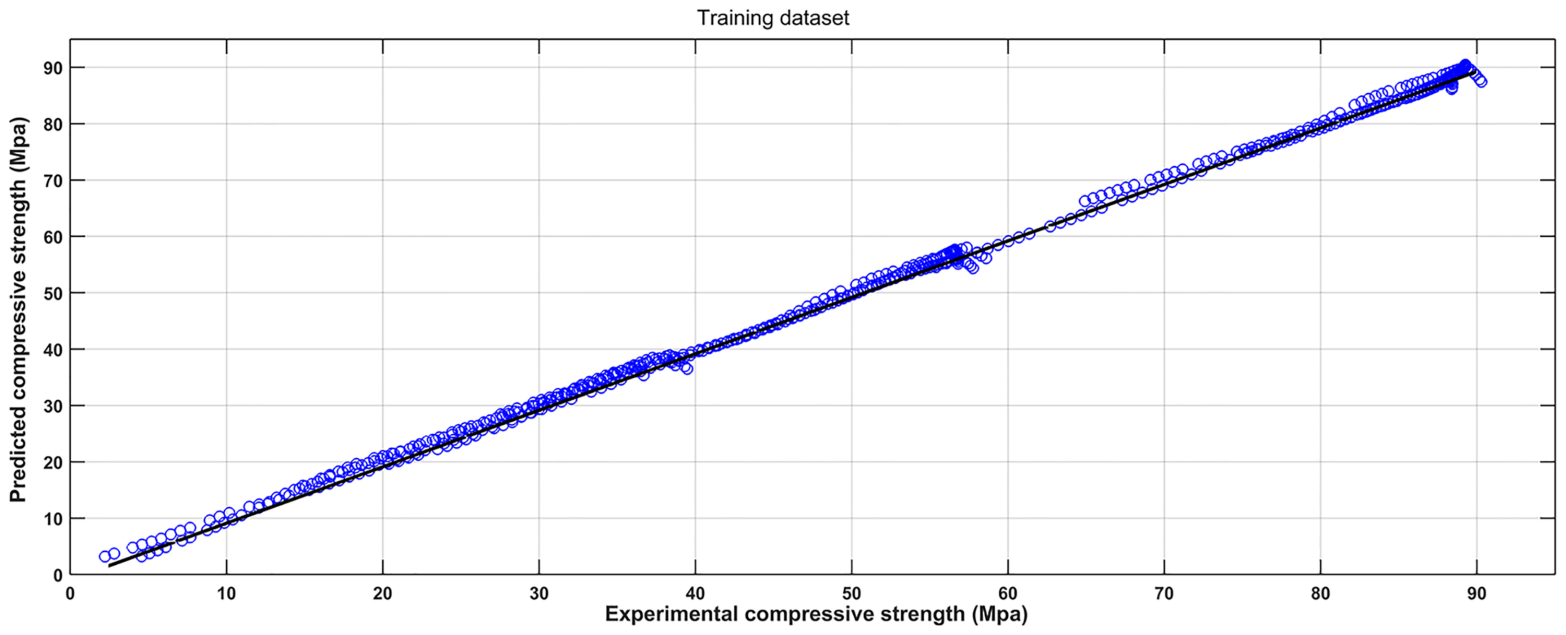
Observed versus predicted compressive strength produced by ANN method (Training dataset).

**Fig 12:**
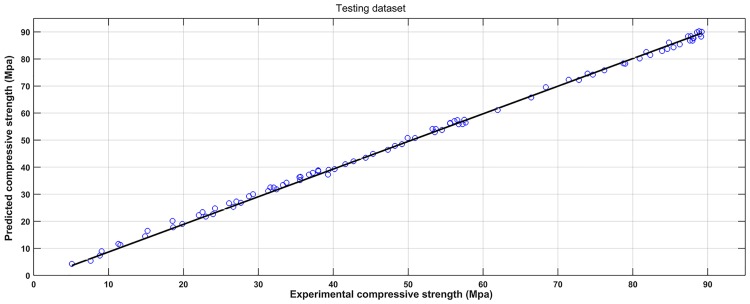
Observed versus predicted compressive strength produced by ANN method (Testing dataset).

The observed versus predicted compressive strength calculated by the ANN model are shown in Figs 11 and 12, and the performance measurement results of the two models are shown in Table 5. For the ANN model, the R^2^ of training data and testing data were 0.9982 and 0.9975, respectively. The MAE of training data and testing data were less than 5.2 MPa. The MAPE of training data and testing data were less than 5.9%. The RMSE of training data and testing data were less than 6.6 MPa. These results indicate that the ANN model is also in the good prediction of concrete compressive strength. The R^2^ is lower, while the RMSE, MAE and MAPE are higher for ANN model compared to the SVM model, indicating that the performance of the SVM model is better than ANN model. The reason is that BP algorithm was used in this study to find suboptimal solutions being trapped in local minimums. Moreover, the number of hidden layer neurons is estimated by the trial and error procedure, and number of neurons in input layer is equal to the number of input variables. Thus, the training iterations may force ANN model to over train, and then affect the predicting capabilities. But for the SVM model, which objective is to construct a hyper plane that lies ‘‘close” to as many of the data points as possible, it can achieve good generalization ability by minimizing the regularized risk function as the main parameters c and g were obtained by a *K*-fold crossover algorithm.

**Table 5:** Performance measurement results of models.

Model	Training dataset				Testing dataset			
	R^2^	MAE(MPa)	MAPE(%)	RMSE(MPa)	R^2^	MAE(MPa)	MAPE(%)	RMSE(MPa)
**SVM**	0.9994	2.16	3.32	2.68	0.9991	3.02	3.71	3.56
**ANN**	0.9982	4.25	5.06	5.32	0.9975	5.19	5.87	6.53

### 4.7 Prediction performance for corroded samples

**Fig 13:**
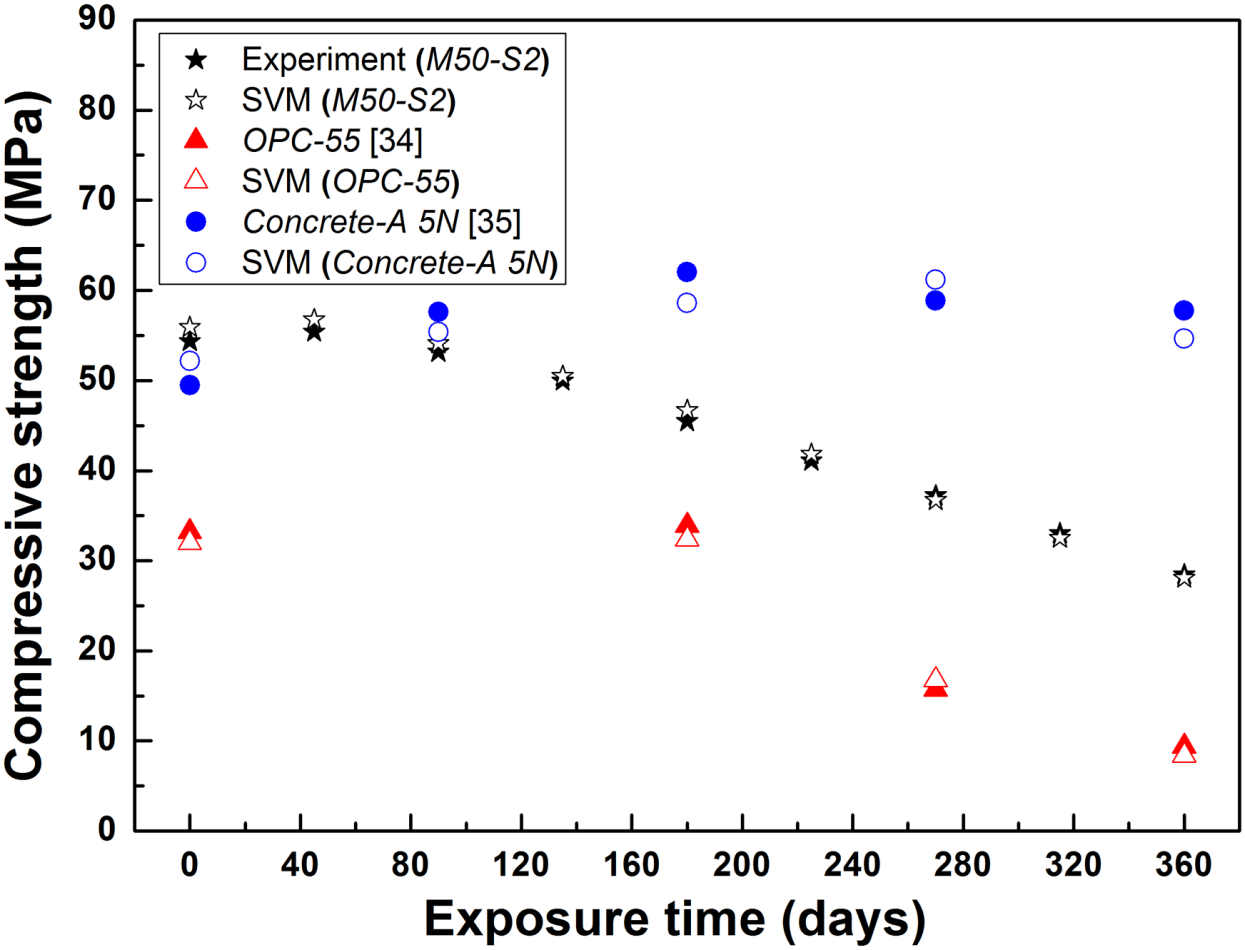
Comparison between predicted values and experimental results. *M50-S2*: cement mortar with w/c ratio of 0.50 in the accelerated degradation test; *OPC-55*: ordinary Portland cement mortar with w/c ratio of 0.55; *Concrete-A 5N*: concrete of Type A exposed to artificial sea water.

The SVM model was further verified by testing it using the experimental data of the compressive strength of cement-based materials deteriorated in sulfate and seawater. A group of experimental data from the accelerated degradation test in this study and reference in the literature [34, 35] were used. Fig 13 shows the comparison between the predicted and experimental compressive strength results. *M50-S2* means cement mortar with w/c ratio of 0.50 in the accelerated degradation test; *OPC-55* means ordinary Portland cement mortar with w/c ratio of 0.55; *Concrete-A 5N* means the concrete of Type A exposed to artificial sea water with concentration of 5 times of normal simulated sea water.

Results show that the predicted compressive strength of concrete from the SVM model matched well with the experimental values of reference [35], while the predicted compressive strengths of mortars from the SVM model matched much better with the experimental values of the accelerated degradation test in this study and reference [34], indicating that SVM can be potentially used as an effective method to predict the compressive strength of cement-based materials in harsh environment. The performance of these predicted compressive strengths is different, the reason is that, besides the exposure time, w/c ratio and sulfate ions, the predicted compressive strength of *Concrete-A 5N* would also be affected by aggregate content and magnesium ions content.

## Conclusions

In this study, a prediction model of mortar compressive strength was established by SVM. A total of 638 sample data collected from the experimental test were used to develop the SVM model for predicting compressive strength. The SVM model was first calibrated and then verified using the experimental data from corroded concrete samples. Conclusions can be drawn as follows:

1. The compressive strength values of all mortar specimens increased and then decreased gradually when the specimens were degraded by sodium sulfate solutions. The experimental results show that, after degraded by the same concentration of solution, the greater the w/c ratios, the larger the descent rate of compressive strength.
2. The sensitivity analysis results show that the main factors influencing the prediction of mortar compressive strength were exposure time, w/c ratio and sulfate concentration.
3. The predicted compressive strengths from the developed SVM model matched well with the experimental values, indicating that the SVM model can be potentially used to for predicting the compressive strength of cement-based materials servicing in harsh environments. Compared to the ANN model, the performance of SVM model is better.

## Supporting information

S1 File Experimental dataset. w/c: water-cement ratio, C: cement content, %; W: water content, %; S: sand content, %; SO_4_^2-^, sulfate concentration, wt%; Wet-T, wetting temperature, °C; Wet-t: wetting time, h; Dry-T, drying temperature, °C; Dry-t: drying time, h; Exp-t: exposure time, days; Fm: ultimate compressive strength, MPa.
(XLSX)
